# Fitness characteristics of the malaria vector *Anopheles funestus* during an attempted laboratory colonization

**DOI:** 10.1186/s12936-021-03677-3

**Published:** 2021-03-12

**Authors:** Halfan S. Ngowo, Emmanuel E. Hape, Jason Matthiopoulos, Heather M. Ferguson, Fredros O. Okumu

**Affiliations:** 1 Department of Environmental Health and Ecological Sciences, Ifakara Health Institute, P.O. Box 53, Ifakara, Tanzania; 2 Institute of Biodiversity, Animal Health and Comparative Medicine, University of Glasgow, Glasgow, G12, 8QQ UK; 3 School of Public Health, University of the Witwatersrand, 1 Smuts Avenue, Braamfontein, 2000 Republic of South Africa; 4 School of Life Science and Bioengineering, Nelson Mandela African Institution of Science and Technology, P.O. Box 447, Arusha, Tanzania

## Abstract

**Background:**

The malaria vector *Anopheles funestus* is increasingly recognized as a dominant vector of residual transmission in many African settings. Efforts to better understand its biology and control are significantly impeded by the difficulties of colonizing it under laboratory conditions. To identify key bottlenecks in colonization, this study compared the development and fitness characteristics of wild *An. funestus* from Tanzania (FUTAZ) and their F_1_ offspring during colonization attempts. The demography and reproductive success of wild FUTAZ offspring were compared to that of individuals from one of the only *An. funestus* strains that has been successfully colonized (FUMOZ, from Mozambique) under similar laboratory conditions.

**Methods:**

Wild *An. funestus* (FUTAZ) were collected from three Tanzanian villages and maintained inside an insectary at 70–85% RH, 25–27 °C and 12 h:12 h photoperiod. Eggs from these females were used to establish three replicate F_1_ laboratory generations. Larval development, survival, fecundity, mating success, percentage pupation and wing length were measured in the F_1_ -FUTAZ offspring and compared with wild FUTAZ and FUMOZ mosquitoes.

**Results:**

Wild FUTAZ laid fewer eggs (64.1; 95% CI [63.2, 65.0]) than FUMOZ females (76.1; 95% CI [73.3, 79.1]). Survival of F_1_-FUTAZ larvae under laboratory conditions was low, with an egg-to-pupae conversion rate of only 5.9% compared to 27.4% in FUMOZ. The median lifespan of F_1_-FUTAZ females (32 days) and males (33 days) was lower than FUMOZ (52 and 49 for females and males respectively). The proportion of female F_1_-FUTAZ inseminated under laboratory conditions (9%) was considerably lower than either FUMOZ (72%) or wild-caught FUTAZ females (92%). This resulted in nearly zero viable F_2_-FUTAZ eggs produced. Wild FUTAZ wings appear to be larger compared to the lab reared F_1_-FUTAZ and FUMOZ.

**Conclusions:**

This study indicates that poor larval survival, mating success, low fecundity and shorter survival under laboratory conditions all contribute to difficulties in colonizing of *An. funestus*. Future studies should focus on enhancing these aspects of *An. funestus* fitness in the laboratory, with the biggest barrier likely to be poor mating.

**Supplementary Information:**

The online version contains supplementary material available at 10.1186/s12936-021-03677-3.

## Background

Malaria transmission in Africa is dominated by species in the *Anopheles gambiae* and *Anopheles funestus* species complexes. Control of these vectors has been the primary driver of malaria reduction since 2000 [1, 2], and requires thorough understanding of their ecology, behaviours and transmission potential [3–9]. Laboratory colonies of *An. gambiae *sensu lato (*s.l.*) have been an invaluable resource for research by enabling experimental studies under controlled conditions. Such colonies have facilitated the characterization of insecticide resistance [10–12], genetics [13, 14]), immunity [15, 16] and key vector demographic profiles [17–19]. Mosquitoes generated from laboratory colonies are also extensively used for semi-field bioassays [4, 20, 21].

In contrast to *An. gambiae s.l*., *An. funestus s.l.* has proven extremely difficult to colonize and maintain under laboratory conditions. The *An. funestus* species complex group consists of at least 13 known species*: Anopheles aruni, Anopheles brucei, Anopheles confusus, Anopheles funestus *sensu stricto (*s.s*.), *Anopheles funestus*-like, *Anopheles fuscivenosus*, *Anopheles leesoni, Anopheles longipalpis* type A, *Anopheles longipalpis* type C, *Anopheles parensis*, *Anopheles rivulorum*, *Anopheles rivulorum-*like and *Anopheles vaneedeni* [22–25]. These species vary in vectorial capacity [26], with only *An. funestus s.s.* thought to play a significant role in malaria transmission [27, 28]. Others, such as *An. rivulorum,* have been reported as minor vectors in Kenya [29], Tanzania [30] and *An. vaneedeni* in South Africa [31].

Colonization of *An. funestus s.s.* has however been problematic, and only two strains have been successfully colonized from wild populations despite several attempts. Both strains were colonized at the Vector Control Reference Laboratory (VCRL) in the National Institute for Communicable Diseases, South Africa, from populations in Angola (FANG) and Mozambique (FUMOZ) [32, 33]. The FUMOZ strain also maintained at other laboratories worldwide, including in Cameroon and in the UK [12], as well as in Tanzania. Several attempts have been made to colonize new *An. funestus* strains [34] from wild populations, but methods used to establish FUMOZ and FANG have not been successful elsewhere [35], including when attempted in the same wild populations where FUMOZ was originally derived (Coetzee, pers. commun.). This inability to repeatedly colonize and establish *An. funestus* in laboratories is responsible for the more limited understanding of the biology of this species compared to other vector species.

Several factors may account for the difficulty of colonizing *An. funestus*. Chief amongst these is eurygamy, the inability of an insect to mate in flight [36, 37]. Eurygamic species are difficult to colonize because they do not exhibit natural mating behaviours, such as swarming [38], under insectary conditions [36, 39, 40]. Many *Anopheles* mosquitoes mate naturally in aerial swarms [41–43] or, to a smaller extent, indoors [44]. Whilst *An. gambiae* will mate readily in the laboratory [4], wild and F_1_ progeny of *An. funestus* rarely swarm inside cages. Although mating is hypothesized to be the main barrier to *An. funestus* colonization, other factors cannot be ruled out due to incomplete or absence of reporting on other aspects of their life history and fitness during attempted colonization. Therefore, it is crucial to comprehensively evaluate how all aspects of *An. funestus* life history, development and demography respond to standard methodologies for colonization in order to identify where modifications should be focused.

Malaria eradication in most African settings will require a more detailed understanding of the basic biology and ecology of vectors of residual transmission, and which additional strategies will most effectively target them. Historically, most research on African malaria vectors has also been concentrated on the *Anopheles gambiae* s.l. species group. *Anopheles funestus* has also recognized as an important vector in many African settings [26, 27, 45]; and is now the dominant source of transmission in many areas following the decline of *An. gambiae* [27, 28, 46]. However in contrast to *An. gambiae s.l.*, much less is known about the ecology and fundamental biology of *An. funestus s.l.* This knowledge gap is due to a range of factors including the more cryptic nature of it larval habitats and most notably the difficulties with colonizing it. Given the growing prominence of this species in mediating residual transmission across Africa, there is a clear need to overcome these obstacles as required to guide the development and implementation of more effective control strategies.

To address these knowledge gaps, the fitness and behavior of wild *An. funestus* from Tanzania and their offspring (defined as “FUTAZ”, i.e. *An. funestus* from Tanzania), were quantified during repeated colonization attempts under standard laboratory conditions. From previous studies, it is known that colonization of this species using standard approaches is difficult. The first step in optimizing this process is to understand which aspects of *An. funestus* life-history and fitness are most impaired during colonization, and thus target modifications appropriately. To assess this, a detailed measurement of the fitness and life-history of wild and F_1_ *An. funestus* were conducted during repeated laboratory colonization attempts. Fitness measures of individuals in this nascent colony were compared to those of a stable *An. funestus* colony (FUMOZ) to identify the key barriers that hinder successful colonization of this species. The term “fitness trait” refers to measures of mating success (insemination status), fecundity (number of eggs produced), adult body size and survival (larval and adult). Insights gained will guide future research to overcome barriers to colonizing *An. funestus*, and also increase knowledge on this important vector and its control.

## Methods

### Study area

**Fig. 1:**
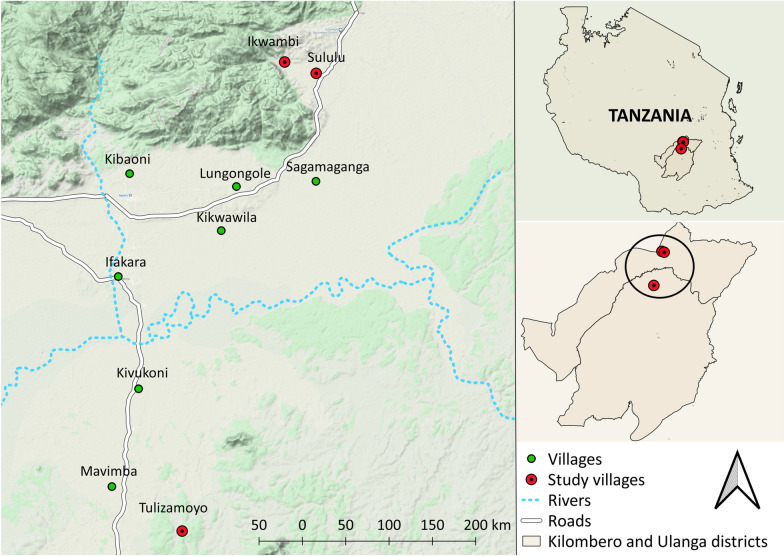
A map of study area showing the location of the villages where *Anopheles funestus* *s.l*. females (wild-FUTAZ) were sampled for colonization experiments. (Kindly prepared by *Najat Kahamba*)

Wild *An. funestus s.l* adults were collected from three villages (Tulizamoyo, Ikwambi and Sululu) in Kilombero (8.1539ºS, 36.6870ºE) and Ulanga (8.3124ºS, 36.6879ºE) districts in Tanzania (Fig. 1). The villages were selected because of their high abundance of *An. funestus s.l.*, of which > 93% are known to be *An. funestus s.s.* [47]. Wild-caught females were transported to the Ifakara Health Institute and used in experiments at the vector biology & control laboratory, the VectorSphere at Ifakara (Fig. 1).

### Mosquito sampling

Five houses were selected for mosquito collection in each village. Mosquito collections were conducted for one week in each village in 2019 (Tulizamoyo: 17–23rd June; Ikwambi: 8–16th July and Sululu: 1–10th September). Due to collection logistics and space limitation in the VectorSphere, only one set of experiments (i.e. with mosquitoes from just one village) was done at a time. Trapping was done using CDC light traps [48, 49] that were set from 6 pm to 6 am for five consecutive nights per village (yielding ~ 200–300 female *An. funestus s.l.* per week). Light traps were fitted with larger catch bags to help keep mosquitoes alive without desiccation until morning. Every morning, live female *An. funestus s.l.* were aspirated from the catch bags into netted cages (30 × 30 cm), provided with 10% glucose solution and brought to the VectorSphere for blood-feeding and further rearing. Inside the VectorSphere mosquitoes were kept under standard conditions of 70–85% RH, 25–27 °C and a 12 h:12 h photoperiod.

### Laboratory maintenance and fitness measurements for FUTAZ mosquitoes

**Fig. 2:**
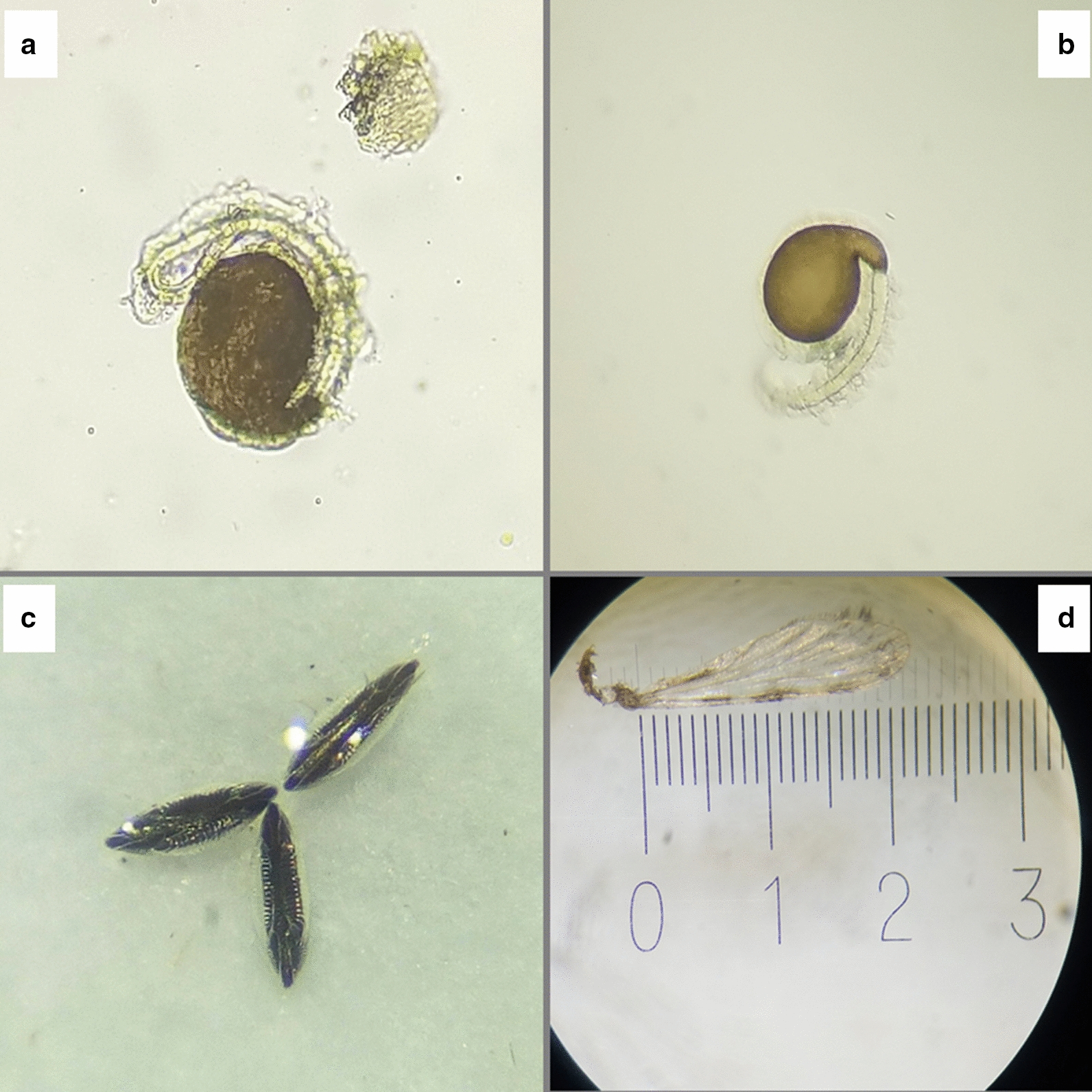
Microscopic images of spermathecae showing the (**a**) presence of the spermatozoa as the confirmation for insemination and (**b**) absence of spermatozoa suggesting non-inseminated, (**c)** Egg structure of the female *Anopheles funestus s.s.* as seen under microscope, a quick and cheap method of species distinction within *Anopheles funestus* group during colonization instead of standard Polymerase Chain Reaction (PCR) and (**d)** the wing measurement under microscope showing the apical notch and the auxiliary margin

Once in the VectorSphere, wild female of *An. funestus s.l.* were given an initial blood meal from a chicken for a maximum of 30 min (from 6:30 pm) inside cages covered with dark cloth. After this first blood meal, mosquitoes were left in the cage until the next morning when their feeding success was recorded by visual observation. Those with a distended, red abdomen were considered to be fully engorged and transferred into individual oviposition cups for egg laying (Additional file 1: Fig. S1). Cotton pads soaked in 10% glucose solution were placed onto the top netting over the cups for additional nutrition. After three days, a small amount of water (~ 5 ml) was put in each cup to stimulate oviposition. Cups were then inspected daily to record if and when eggs were laid, and dead mosquitoes were removed. Mosquitoes that did not lay eggs after 12 days were killed by freezing for 10 min and later dissected to assess insemination. The terminalia and last abdominal segment (segment IX) were cut-open in distilled water to expose the spermathecae. Slide mounts of spermathecae were inspected using a microscope at 400× magnification for presence of sperm (Fig. 2a&b).

F_1_ eggs from wild-caught *An. funestus s.l.* were identified to species-level based on their morphological characteristics [50]. Eggs were observed under a Stereo Microscope, and sub-samples of emergent adults verified by PCR [51]. All eggs morphologically confirmed as belonging to *An. funestus s.s.,* were retained for use in subsequent colonization and life history experiments and defined as F_1_-FUTAZ (Fig. 2c). Here, all F_1_-FUTAZ eggs were pooled and redistributed into a series of 5L round plastic basins (30 cm diameter, filled to 3.3 cm with tap water, replaced every 2 days) at approximate densities of 400–600 eggs per basin and left to hatch. There were a total of 12 replicate basins set up for each of the 3 independent colonization experiments (1 per study village). From here onwards, the term *An. funestus* refers specifically to *An. funestus s.s*.

Larvae were fed daily with a pinch (approx. 0.36 g) of a mixture of finely crushed dog biscuits and brewer’s yeast at a ratio of 3:1 [32]. Basins were checked daily to record egg hatching and larval survival. Pupae that emerged over three consecutive days were recorded and retained for use in F_1_ adult mosquito fitness experiments. Female: male ratio was also recorded at this stage by looking at the genital lobe shape (i.e. at the end of the pupae abdominal segments just below the paddles, also males tends to be smaller than females [52, 53]. Pupae were placed in a single cage (30 × 30 cm) and monitored until emergence (1–2 days). A total of three cages were set up for each of the three independent colonization experiments.

F_1_ FUTAZ females were offered their first human blood meal five days post-emergence, on the same day and time as females from the FUMOZ colony their fitness was compared with (see below). Females were provided with additional blood meals every five days. The rationale for blood feeding every 5 days was to estimate the survival under conditions where they had access to blood meals at a frequency similar to that expected in the wild. Studies indicate that the gonotrophic cycle length of *An. funestus* ranges between 2 and 5 days [54, 55]; with 5 being selected here due to practical considerations. From the first blood meal onwards, cages were inspected daily to record and remove all dead mosquitoes. A total of three cages were used as replicates in each of the 3 independent colonization experiments. All the dead females were dissected to assess insemination status. Random sub-samples of F_1_ females were selected after the second blood meal and moved into individual oviposition cups to measure their egg production.

### Laboratory maintenance and fitness measurements for FUMOZ mosquitoes

In July 2018, eggs from the FUMOZ *An. funestus* colony were obtained from the VCRL laboratory in South Africa and used to establish a colony within the VectorSphere, at Ifakara Health Institute in Tanzania. The founder FUMOZ colony at VCRL has been maintained since 2000 [32]. At IHI, the FUMOZ colony was maintained for four generations before starting these experiments. This colony was kept under the same insectary conditions (70–85% RH, 25–27 °C and 12 h:12 h photoperiod) in the VectorSphere and same feeding regime as described above for F_1_-FUTAZ. In this study, the following fitness variables were measured in FUMOZ for comparison with FUTAZ: number of eggs laid per mosquito (fecundity), proportion of eggs hatched, wing lengths, proportion of adult female inseminated, proportion of larvae survived, larval development period, and proportion of pupae emerged as adult, female: male sex ratio at pupae stage and number of days survived by adult females. The definition of all fitness traits measured, and the colonies in which they were made were given in (Additional file 1: Table S1).

### Mosquito wing size measurements

The wing lengths of all female *An. funestus* (Wild FUTAZ, F_1_-FUTAZ and FUMOZ) were measured and used as a proxy for their body size. One wing was removed from each mosquito and placed onto a drop of water on a microscope slide. The wing lengths were measured using the micrometer ruler under a microscope (50 mm micrometer scale in 0.1 mm divisions, 70 mm × 20 mm × 3 mm) [56]. Measurements were taken from the apical notch to the auxiliary margin, excluding the wing fringe (Fig. 2d).

### Ethics

This study was approved by Ifakara Health Institute Institutional Review Board (Ref. IHI/IRB/No: 007-2018) and the Medical Research Coordinating Committee (MRCC) at the National Institute for Medical Research-NIMR (Ref: NIMR/HQ/R.8a/Vol.IX/2895). Permission to publish was obtained from NIMR (Ref: NIMR/HQ/P.12 VOL XXXI/57). Individual verbal and written consent was also obtained from household owners where CDC light traps were placed for collecting mosquitoes and verbal consent for the arm-feeders in the insectary*.*

### Statistical analysis

Data analyses were conducted using R statistical software version 3.5.0 [57]. Mean values were estimated for *An. funestus* fitness traits (Additional file 1: Table S1 in SI), and how these variables differs between FUTAZ and FUMOZ strains. Where possible, fitness traits (i.e. wing length and proportion inseminated) were also compared among the wild FUTAZ, F_1_-FUTAZ and FUMOZ. Additional analysis was conducted to assess the relationship between female body size and fecundity (number of eggs produced) in wild FUTAZ and FUMOZ.

Generalized linear mixed models (GLMM) implemented in *lme4* package [58] were used to estimate mean values of fitness traits in wild and F_1_-FUTAZ and FUMOZ strains. Fecundity, the number of eggs laid per mosquito, was modeled as a Poisson variate and wing length was included in the model as fixed effects. For proportion data (here, emergence, insemination, larval survival and sex ratio, (Additional file 1: Table S1) were modeled as a binomial variate with strain used as fixed effects while replicates as random effects. Wing length and strain were also used as fixed effect when assessing insemination. Wing length was modeled as a Gamma variate with an inverse link function, incorporating strain as fixed effect. Tukey’s post-hoc tests were used to assess the means differences for different fitness measurements.

Survival analyses were done using a Cox proportional hazard model using the *survival* package [59] to assess the odds of mortality for males and females for each strain and for females of the two strains of *An. funestus* (F_1_-FUTAZ vs. FUMOZ). Here, the response variable was the death occurring on each day of observation, while strains and sex were included as explanatory variables. In the analysis of F_1_-FUTAZ, site of collection was included as a random effect by fitting a frailty function [60, 61] using a Gamma distribution. Separate analyses were performed for each strain except when the differences between strains were investigated. Log likelihood ratio tests (LRT) were used to test the significance of each variable of interest in all models. All figures were produced using *ggplot2* [62] and *survminer* [63] R packages.

## Results

A total of 1,130 adult females of the wild-FUTAZ strain were collected from the three different villages, Tulizamoyo (n = 332); Ikwambi (n = 425); Sululu (n = 373). More than two-thirds (n = 804) of these successfully fed when offered a blood-meal in the insectary, of which 39% (n = 316) laid eggs in the insectary.

### Mosquito wing lengths, mating status, fecundity and pupation

**Fig. 3:**
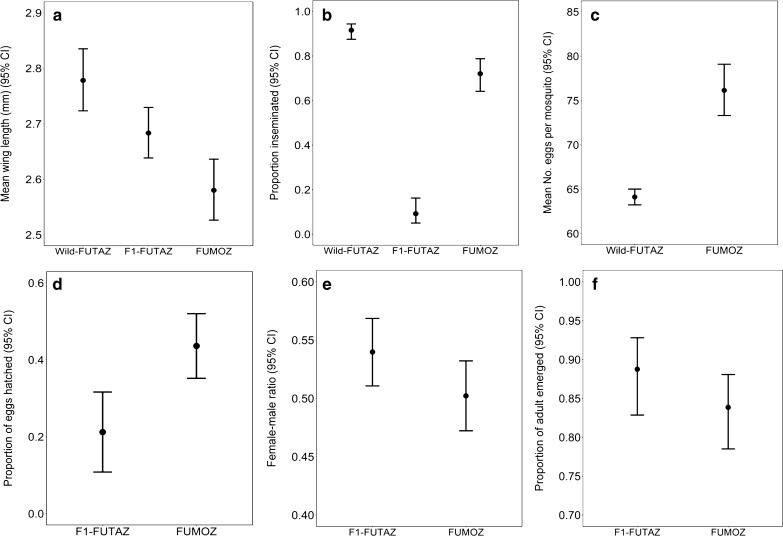
Showing *Anopheles funestus* (**a**) wing sizes; (**b)** female inseminated, (**c)** fecundity, (**d)** eggs hatched, (**e)** sex ratio, (**f)** adult emerged for Wild-FUTAZ, F_1_-FUTAZ and FUMOZ strain

*Anopheles funestus* wing lengths varied significantly between groups (*χ*^2^ = 14.97, p < 0.001, Fig. 3a). A Tukey’s *post-hoc* test showed that the wild-collected FUTAZ were larger than lab reared F_1_-FUTAZ (z = 3.23, p < 0.01, Fig. 3a) and FUMOZ (z = 2.52, p < 0.05, Fig. 3a). There was no difference in wing lengths between the two laboratory-reared strains, FUMOZ and F_1_-FUTAZ (z = 1.43, p = 0.303, Fig. 3a). The wing lengths of F_1_-FUTAZ (*χ*^2^ = 10.4, p < 0.01) but not FUMOZ (*χ*^2^ = 0.123, p = 0.688) were positively associated with insemination status. Furthermore, the proportion inseminated varied significantly between strains (*χ*^2^ = 177.2, p < 0.001, Fig. 3b), and the two generations of FUTAZ (*χ*^2^ = 172.3, p < 0.001, Fig. 3b). Insemination was considerably lower in F_1_-FUTAZ (9%) compared to wild caught FUTAZ females (92%) and FUMOZ (72%; Fig. 3b).

**Fig. 4:**
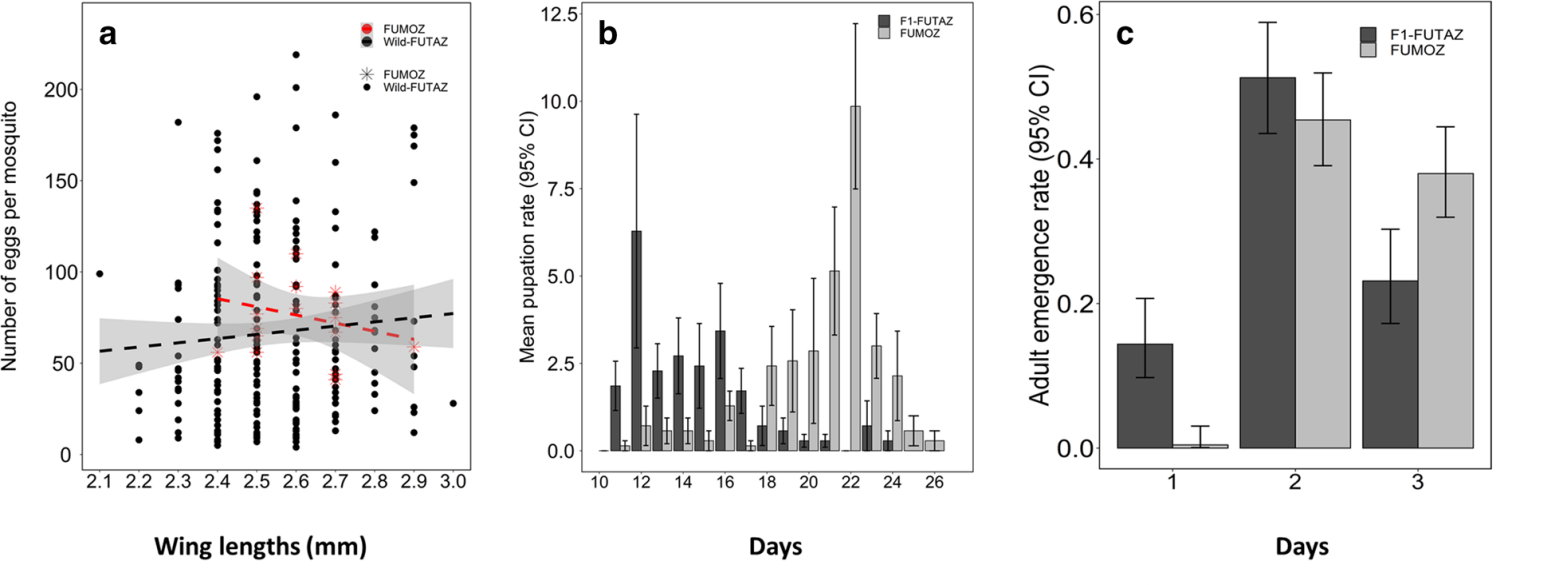
*Anopheles funestus* (**a**) relationship between mosquito body sizes and number of eggs produced per *Anopheles funestus* mosquito, (**b)** larvae period and (**c)** Pupae period

Fecundity varied between strains (*χ*^2^ = 66.54, p < 0.001) with FUMOZ females producing significantly more eggs than wild-FUTAZ (Fig. 3c, Table 1). FUMOZ clutch size ranged from 41–137 eggs while wild-FUTAZ clutch sizes range from 3–236 eggs. The proportion of eggs hatched into 1^st^ instar larvae was 21% [95% CI; 10.8, 31.6] in F_1_-FUTAZ and 44% [35.2, 52.0] in FUMOZ (Fig. 3d). No eggs were produced by F_1_-FUTAZ. The impact of wing length on fecundity varied between strains (*χ*^2^ = 62.57, p < 0.001, Fig. 4a).

**Table 1:** Relative odds (OR) and means of insemination, sex ratio, pupation and adult emergence for different strains of *Anopheles funestus*, number in brackets are 95% confidence intervals with their respective p-values

Variable	Strain	Mean ± 2SE	OR [95% CI]	P-value
%Insemination	Wild-FUTAZ	91.5 ± 1.30	1	
	F_1_-FUTAZ	9.2 ± 4.99	0.009 [0.004, 0.020]	< 0.001
	FUMOZ	72.0 ± 5.16	0.24 [0.13, 0.42]	< 0.001
%Sex ratio (F/M)	F_1_-FUTAZ	53 ± 4.0	1	= 0.049
	FUMOZ	50 ± 2.9	0.86 [0.74, 0.99]	
%Larval survival	F_1_-FUTAZ	5.3 ± 3.5	1	< 0.001
	FUMOZ	27.4 ± 12.5	12.05 [4.27, 34.03]	
%Adult emergence	F_1_-FUTAZ	88.8 ± 7.35	1	= 0.174
	FUMOZ	81.6 ± 8.90	0.66 [0.36, 1.20]

The median larval development period from 1^st^ instar larvae to pupation was 22, IQR: 21–23 days in FUMOZ, and only 13, IQR: 11–14 days in F_1_-FUTAZ (Fig. 4b). Overall, the proportion of eggs surviving to pupation was 5.87% in F_1_-FUTAZ and 27.4% in FUMOZ, reflecting significant variation between strains (*χ*^2^ = 11.28, p < 0.001, Table 2). The sex ratio (females: males) in pupae varied marginally between strains (*χ*^2^ = 3.89, p = 0.049, Fig. 3e), with a slightly higher proportion of females in F_1_-FUTAZ compared to FUMOZ (Table 2). The proportion of adults that emerged from pupae was similar in F_1_-FUTAZ and FUMOZ (*χ*^2^ = 1.91, p = 0.167, Fig. 3f), with most adults emerging on the second day of pupation (Fig. 4c).

**Table 2:** Relative risk (RR) and means of fecundity for different strains of *Anopheles funestus*, number in brackets are 95% confidence intervals

Variable	Strain	Mean ± 2SE	RR [95% CI]	P-value
Fecundity	Wild-FUTAZ	64.1 ± 5.26	1	
	FUMOZ	76.1 ± 7.61	0.84 [0.81, 0.88]	< 0.001

### Adult survival

**Fig. 5:**
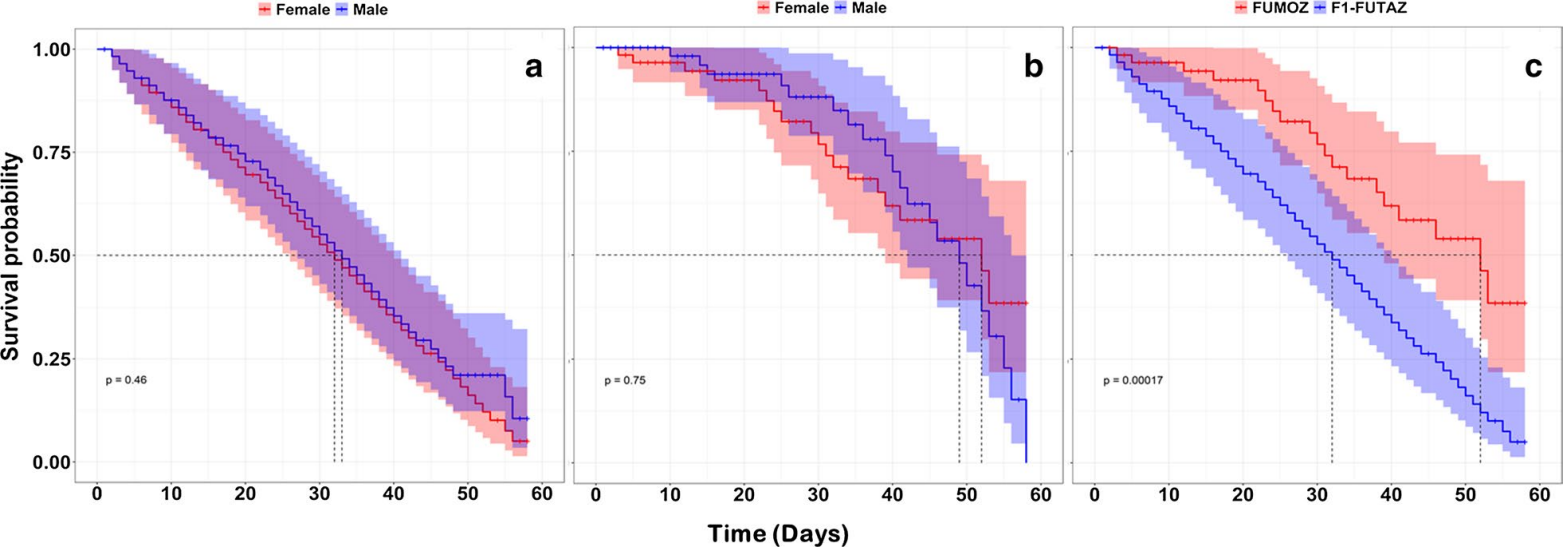
Survival of males and females of (**a**) *Anopheles funestus* (F_1_-FUTAZ) and (**b**) *Anopheles funestus* (FUMOZ) when feed after every 5 days, (**c**) females of both F_1_-FUTAZ and FUMOZ. Lines represent the survival function as estimated from fitting the Cox proportion hazard model and shaded area express 95% CI. Dotted grey horizontal and vertical lines show the median survival days

The median survival of adult female F_1_-FUTAZ was 32 (IQR: 26, 40) and 33 days (IQR: 27, 41) for males (Fig. 5a). In FUMOZ, the median survival for females was 52 days (IQR: 39, 56) and 49 days (IQR: 42, 56) for males (Fig. 5b). There was no difference in the survival of males and females within either strain, F_1_-FUTAZ (p = 0.468, Table 3) and FUMOZ (p = 0.752, Table 3). However, restricting analysis to adult females, survival was significantly lower in F_1_-FUTAZ than FUMOZ (p < 0.001, Table 3, Fig. 5c). Likewise, adult males survival was significantly lower in F_1_-FUTAZ than FUMOZ (p < 0.01, Table 3).

**Table 3:** Hazard Ratio and median values of the adult survival between males and females of the F_1_-FUTAZ and FUMOZ, associated p-values indicate the significance difference of sex and species on the number of days survived by F_1_-FUTAZ and FUMOZ strains

Strain	Sex	Median [IQR]	HR [95% CI]	P-value
F_1_-FUTAZ	Female	32 [26, 40]	1	= 0.468
	Male	33 [27, 41]	0.89 [0.65, 1.24]	
FUMOZ	Female	52 [39, 56]	1	= 0.752
	Male	49 [42, 56]	1.11 [0.59, 2.06]	
Female	FUMOZ	52 [39, 56]	1	< 0.001
	F_1_-FUTAZ	32 [26, 40]	2.63 [1.55, 4.46],	
Male	FUMOZ	49 [42, 56]	1	< 0.01
	F_1_-FUTAZ	33 [27, 41]	2.05 [1.22, 3.45]	

## Discussion

Limited understanding of mosquito biology and ecology poses a challenge for the development of effective vector control approaches. Laboratory colonization of target species provides an opportunity to address these knowledge gaps by facilitating detailed investigation of vector biology under controlled conditions where experimental manipulation is possible. Here the fitness traits of *An. funestus* during colonization attempts from a wild population in Tanzania were characterized to identify the bottlenecks that make this species so difficult to colonize. This is the first documentation of fitness constraints during attempted colonization of this species and the first report of attempted colonization of *An. funestus* from Tanzania.

Consistent with most previous attempts, colonization of this wild *An. funestus* population proved unsuccessful with no offspring being produced from the F_1_ generation. Several life history processes and demographic traits were identified as being impaired when FUTAZ were brought into the laboratory. First, there number of eggs laid by wild FUTAZ when brought in the insectary was lower than in the well-establish FUMOZ line, as were hatching rates, larval survival, mating success and adult female/male survival. F_1_ FUTAZ body sizes were slightly smaller compared to their maternal generation in the wild, but did not differ with the FUMOZ strain indicating this trait is unlikely to predict colonization success. Of all these fitness traits, the primary hurdles to colonization are likely to be the extremely low mating success and larval survival of F_1_ *An. funestus* in the laboratory. Until these fitness traits can be improved under laboratory conditions, the colonization of *An. funestus* is unlikely to be successful and repeatable.

Eggs laid by wild-FUTAZ *An. funestus* had low proportion of hatching compared to those of FUMOZ, though they were both lower than 50%, indicating many were unviable eggs laid by non-inseminated females. Previous studies investigating the impact of different water sources used for larval rearing in an *An. funestus* colony (FUMOZ) indicated that their egg hatching rate can exceed 70% [64, 65]; confirming hatch rates in this study were low. It is known that females of other *Anopheles* species can produce unviable eggs without successful mating, or after mating with sperm-less males [66]. Therefore, poor hatching observed in the nascent strain (FUTAZ) here is likely due to first, the low mating success of *An. funestus* in captivity as has been previously documented in other *Anopheles* species [35], second since the number of eggs laid was very low, the absence of any hatching could be just stochastic effect, lastly, low hatching rate could also be associated with suboptimal temperature in the laboratory relative to the one in the field to which FUMOZ strain might have been well adapted to it but not FUTAZ [67].

The larval development period of F_1_-FUTAZ (11–14 days) was similar to that reported for *An. funestus* in other laboratory settings [64, 65, 68], but faster than FUMOZ development period (21–23 days) observed in this study. The duration of larval development in *An. funestus* (FUTAZ and FUMOZ) observed here were considerably longer than described for *An. gambiae* complex in the laboratory [69]. For example, life table analyses of *An. gambiae* indicate larval development period from eclosion to adult emergence of about 11 days at 27 °C [55, 69–72]. This long larval development period for F_1_-FUTAZ results in a long estimated generation time of 30–33 days from eggs to first oviposition; which is higher than estimated for other African *Anopheles* species [64, 73, 74]. Other life table analyses performed on *An. funestus* colonies estimated a generation time of approximately 33 days in insecticide-resistant (FUMOZ) and susceptible strains (FANG), [65]. As a consequence of this extended period of larval development, the egg to pupa survival was very low; approximately 6% for F_1_-FUTAZ and 27% for FUMOZ. Due to this long larval development and associated high larval mortality, very large numbers of eggs would be required generate modest numbers of adults in the laboratory. Therefore, the fitness and reproductive success of these resulting adults would have to be very high to yield a further generation.

Analysis of wild FUTAZ adults and their F_1_ offspring indicate their fitness is reduced compared to that of a stable *An. funestus* colony (FUMOZ). Wild-FUTAZ *An. funestus* brought into the laboratory laid 16% fewer eggs than the FUMOZ colony, and the F_1_ generation of FUTAZ produced no viable eggs at all. A previous study measuring the fecundity of F_1_ *An. funestus* using Madagascan population reported that this species can lay and average of 56 to 108 eggs per mosquito in captivity [35], which corroborates with 65 and 76 eggs from wild-FUTAZ and FUMOZ, respectively, from the current study. The number of eggs here is consistent to that reported in resistant and susceptible *An. funestus* strains in the laborator*y,* [65].

Mosquito body size is often interpreted as a proxy of their fitness [75–77]. Here, wild FUTAZ were somewhat larger in body size than F_1_-FUTAZ and FUMOZ. Consistent with the hypothesis of body size being an indicator of fitness, wing length was positively correlated with fecundity in the wild population of *An. funestus* (FUTAZ). However, the opposite was seen in the stable FUMOZ strain where wing size was negatively associated with fecundity. Thus, at least in this one stable laboratory colony, large body size in *An. funestus* was not a good indicator of reproductive success. Caution is required in extrapolating fitness differences based on *An. funestus* body size, particularly between field and laboratory strains. Although body size fell between wild-FUTAZ and F_1_-FUTAZ, these mosquitoes were still bigger than the FUMOZ which had the highest fecundity.

The mating success of *An. funestus* from these populations was extremely low in the laboratory, supporting hypothesis that mating is the key bottleneck for the colonization of this species. Compared to wild-FUTAZ, insemination rates in F_1_-FUTAZ were extremely low (9.2% vs. 72%) and insufficient to establish a further generation F_2_-FUTAZ. This poor mating success is likely due eurygamy, the inability of some *Anopheles* species including *An. funestus* to initiate natural swarming behaviour in flight [78, 79]. These findings match those of other studies documenting mating as the major obstacle for successful colonization of *An. funestus* [34, 35, 80]. To overcome this problem, techniques such as forced mating and exposing mosquitoes during sunset to induce swarming have been applied [81, 82]. Other studies have experimented with the use of large cages to stimulate natural mating for *Anopheles,* and simulate sunset which may be crucial cue for mating [83, 84]. However so far these methods have had little or no success over multiple attempts [32, 34]. In the current study, no F_2_-FUTAZ offspring were generated because none of the F_1_-FUTAZ laid viable eggs. Further research on how to induce mating behavior in *An. funestus*, particularly using more realistic semi-field systems, would be of great value. Such studies must focus on both females and males, to determine if males are unwilling to initiate swarming behaviour or not fit enough to do so.

Analysis of adult mosquito survival indicated that the nascent Tanzania colony (F_1_-FUTAZ) had a reduced lifespan compared to stable *An. funestus* colony (FUMOZ). However adult survival in both cases was relatively high (32 median days for FUTAZ and 52 days for FUMOZ); with both strains living well beyond the minimum period required to produce eggs and transmit malaria. Another laboratory study conducted on FUMOZ where adult life span ranged from 39 to 64 days [65]; again much higher than F_1_-FUTAZ here. The shorter life span of FUTAZ relative to FUMOZ may be a result of the stress from the change of environment, or lack of adaption to laboratory conditions. Nevertheless, this F_1_-FUTAZ survived much longer compared to another competent vectors of malaria transmission, *Anopheles arabiensis* and *An. gambiae s.s.* in the laboratory conditions [21]. Previous experiments on parity shows that the median survival of *An. funestus* in the wild is much shorter, ranging from 7 to 10 days in the wild population [85]. Thus, poor adult survival relative to the wild cannot explain the failure of colonization here.

A potential limitation of our study is that the unfed *An. funestus* females were used to seed laboratory colonies, requiring us to blood feed them artificially (on chicken blood) in the laboratory to acquire eggs for the next generation. Thus, the mating status and age of the wild females used for colonization were uncertain and likely variable. An alternative would have been to collect only visibly blood fed females during field collections (these individuals would likely had fed on humans if caught inside houses), and used their eggs to generate the F_1_ generation. This was considered, but given the much lower abundance of blood fed *An. funestus* inside houses compared to the numbers of unfed females that can be obtained in CDC light traps; the latter approach was chosen to ensure sufficiently large samples were obtained for colonization experiments. These wild mosquitoes could not be provided with a human blood meal given their malaria infection status was unknown and they were not adapted to membrane feeding, thus chicken blood was provided. This variation in host blood source could have generated some differences in fitness between strains. However, this is unlikely given that previous studies indicate that human and chicken blood meals generate similar egg production in other African malaria vector species under laboratory conditions (*An. arabiensis* and *An. gambiae* [21, 86]). The F_1_ population, upon which the main fitness indicators were assessed, was fed on human blood. Further investigation is required to confirm whether *An. funestus* fitness is impacted by the type of host blood meals provided and whether there is an optimal diet for laboratory maintenance.

## Conclusions

Laboratory colonies remain fundamental for research on the biology and control of mosquito vectors, by providing a stable and standardized source of mosquitoes for experimental studies. This study provides additional evidence of the intractability of *An. funestus* to colonization. By quantifying a comprehensive range of fitness traits during unsuccessful attempts, this study generates insights into the most important barriers to colonization. Of the range of traits investigated, the primary barrier to colonization was identified as low mating success, compounded further by the slow development and poor survival of the small numbers of larvae produced. Additionally, both the fecundity and adult survival of *An. funestus* offspring from wild parents were reduced under laboratory conditions, but these impacts may have been relatively minor compared to the consequences of poor mating success and poor larval survival. This combination of fitness deficits presents a major challenge for successful colonization and mass rearing of *An. funestus*. To overcome this, future research should focus on enhancing the efficiency these life-cycle processes under insectary conditions. Additionally, the demographic rates estimated from wild and F_1_ -FUTAZ will provide useful baseline information for understanding and modeling *An. funestus* population dynamics in general, and guiding further attempts to its colonization.

## Supplementary Information

**Additional file 1: Table S1.** Descriptions of terms used and strains compared as used in this study. **Table S2.** Total number of mosquitoes for each replicates and sex as used during the survival analysis between two strains (of FUTAZ and FUMOZ). **Figure S1.** Images of: **a)** Series of wooden cages with oviposition cups, each containing a single fully-engorged Anopheles funestus female. Individual cups were used to measure the number of eggs laid by a single mosquito after full blood meal, **b)** Technician counting the number of eggs and measuring the wing sizes of individual mosquitoes which have laid eggs, **c)** Technician aspirating mosquitoes from the rearing cage using mouth aspirator and **d)** bowl contains eggs of *Anopheles funestus*. All experiment was done within the VectorSphere at Ifakara Health Institute.

## Data Availability

The dataset generated by this study is available from the corresponding author upon reasonable request.
