# Supplementary material for: Fitness characteristics of the malaria vector Anopheles funestus during an attempted laboratory colonization
Source: Malar J. 2021 Mar 12;20:148. doi: 10.1186/s12936-021-03677-3 (PMC7955623; doi:10.1186/s12936-021-03677-3)

**Essential fitness characteristics of the malaria vector, *Anopheles funestus*, during an attempted laboratory colonization**

Halfan S. Ngowo^1,2*^, Emmanuel E. Hape^1,2^, Jason Matthiopoulos^2^, Heather M. Ferguson^1,2¥^, Fredros O. Okumu^1,2,3,4¥^

* Corresponding author

¥ Equal supervision role

**Affiliations**

1. Department of Environmental Health & Ecological Sciences, Ifakara Health Institute, P.O. Box 53 Ifakara, Tanzania
2. Institute of Biodiversity, Animal Health and Comparative Medicine, University of Glasgow, G12, 8QQ, United Kingdom
3. School of Public Health, University of the Witwatersrand, 1 Smuts Avenue, Braamfontein 2000, Republic of South Africa
4. School of Life Science and Bioengineering, Nelson Mandela African Institution of Science & Technology, P.O. Box 447, Arusha, Tanzania

**Supplementary Information**

**Table S1: Descriptions of terms used and strains compared as used in this study**

| **Variable measured** | **Strains compared** | **Definition as used in this paper** |
| --- | --- | --- |
| Fecundity | Wild-FUTAZ vs. FUMOZ | Number of eggs laid by a single female mosquito |
| Wing size | Wild-FUTAZ vs. F_1_-FUTAZ vs. FUMOZ | Length (mm) of a wing from apical notch to the auxiliary margin |
| % eggs hatched | F_1_-FUTAZ vs. FUMOZ | Number of eggs hatched as a percentage of the total number of eggs laid |
| % Larvae survival | F_1_-FUTAZ vs. FUMOZ | Number of larvae pupating, as a percentage of all eggs produced per individual female |
| Larval development period | F_1_-FUTAZ vs. FUMOZ | Number of days from 1^st^ instar larvae to pupation |
| Sex ratio | F_1_-FUTAZ vs. FUMOZ | Ration of the number of females to number of males as identified at the pupal stage |
| % Adult emerging | F_1_-FUTAZ vs. FUMOZ | Number of adult emerged as a percentage of pupae stage |
| % Female inseminated | Wild-FUTAZ vs. F_1_-FUTAZ vs. FUMOZ | Number of females found to be inseminated as a percentage of total dissected |
| Adult survival rate | F_1_-FUTAZ vs. FUMOZ | Number of days survived by an adult mosquito in the laboratory |

**Supplementary Information**

**Table S2: Total number of mosquitoes for each replicates and sex as used during the survival analysis between two strains (of FUTAZ and FUMOZ)**

| **Group** | **Male** | **Female** |
| --- | --- | --- |
| Replicate 1 | 59 | 78 |
| Replicate 2 | 29 | 26 |
| Replicate 3 | 31 | 46 |
| Replicate 4 | 65 | 54 |

**Supplementary Information**

**Figure S1:** Images of: **a)** Series of wooden cages with oviposition cups, each containing a single fully-engorged Anopheles funestus female. Individual cups were used to measure the number of eggs laid by a single mosquito after full blood meal, **b)** Technician counting the number of eggs and measuring the wing sizes of individual mosquitoes which have laid eggs, **c)** Technician aspirating mosquitoes from the rearing cage using mouth aspirator and **d)** bowl contains eggs of *Anopheles funestus*. All experiment was done within the VectorSphere at Ifakara Health Institute.


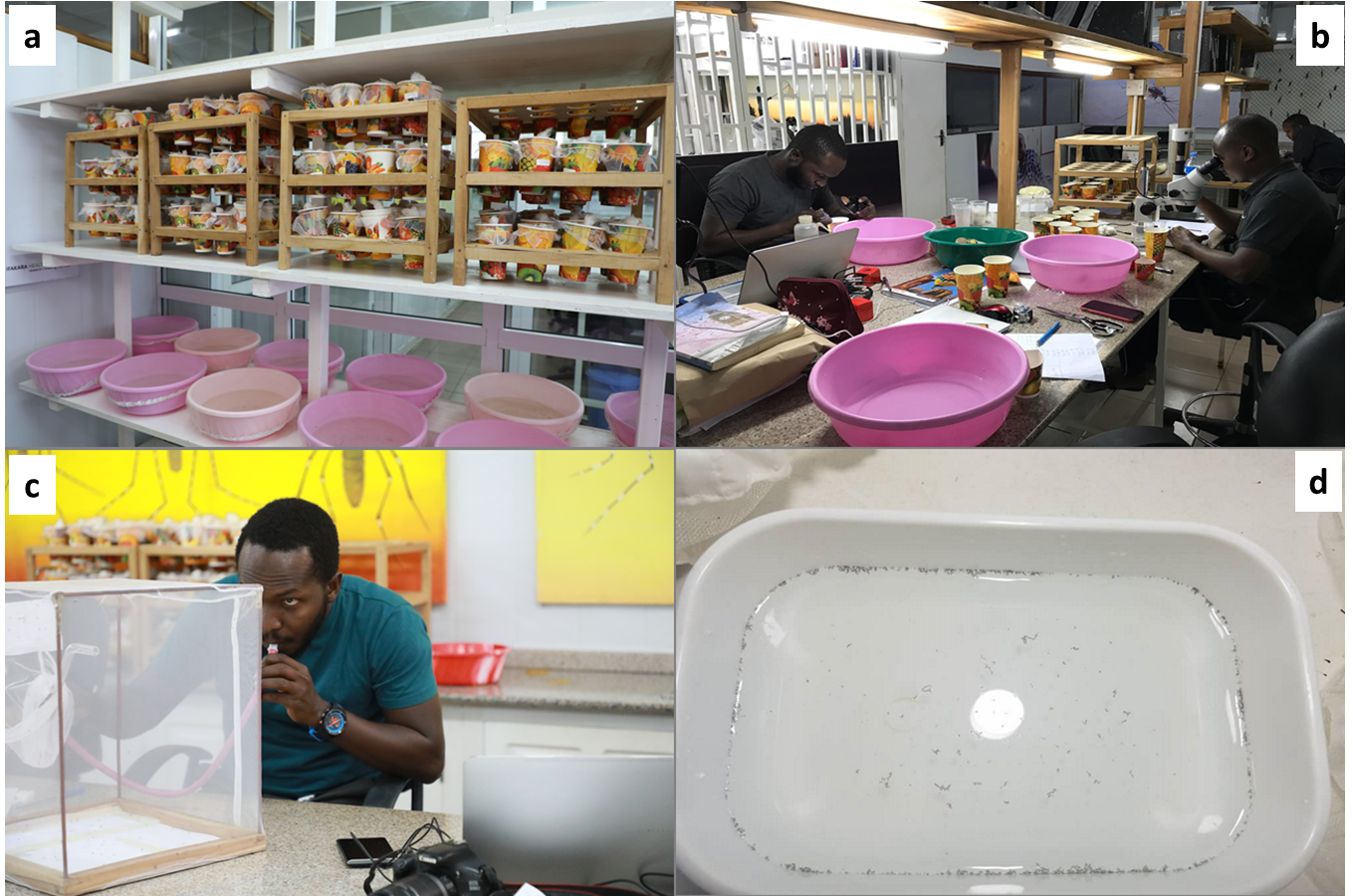

Supplement: Supplementary file 1 — Additional file 1: Table S1. Descriptions of terms used and strains compared as used in this study. Table S2. Total number of mosquitoes for each replicates and sex as used during the survival analysis between two strains (of FUTAZ and FUMOZ). Figure S1. Images of: a) Series of wooden cages with oviposition cups, each containing a single fully-engorged Anopheles funestus female. Individual cups were used to measure the number of eggs laid by a single mosquito after full blood meal, b) Technician counting the number of eggs and measuring the wing sizes of individual mosquitoes which have laid eggs, c) Technician aspirating mosquitoes from the rearing cage using mouth aspirator and d) bowl contains eggs of Anopheles funestus. All experiment was done within the VectorSphere at Ifakara Health Institute. [file 12936_2021_3677_MOESM1_ESM.docx]
